# Real-world platinum selection in immune checkpoint inhibitor-based therapy for recurrent or metastatic head and neck cancer: an exploratory multi-institutional retrospective study

**DOI:** 10.1038/s41598-026-50081-5

**Published:** 2026-04-24

**Authors:** Jumpei Yoshida, Naoki Fukuda, Takumi Ito, Kenji Nakano, Chiyoe Kitagawa, Hiroki Mitani, Shunji Takahashi, Yuji Miura

**Affiliations:** 1 Department of Medical Oncology, Cancer Institute Hospital, Japanese Foundation for Cancer Research, Koto-ku, Tokyo, Japan; 2 Department of Clinical Chemotherapy, Cancer Chemotherapy Center, Japanese Foundation for Cancer Research, Koto-ku, Tokyo, Japan; 3 Department of Medical Oncology, NHO Nagoya Medical Centre, Nagoya, Aichi Japan; 4 Department of Head and Neck Oncology, Cancer Institute Hospital, Japanese Foundation for Cancer Research, Koto-ku, Tokyo, Japan

**Keywords:** Cisplatin, Carboplatin, Pembrolizumab, Immune checkpoint inhibitors, Head and neck cancer, Cancer, Oncology

## Abstract

The KEYNOTE-048 trial established pembrolizumab plus platinum and 5-fluorouracil (5-FU) as the standard first-line treatment for recurrent or metastatic head and neck squamous cell carcinoma (R/M HNSCC). In the present study, the efficacy and safety of cisplatin and carboplatin in combination with pembrolizumab were compared using real-world data. In this retrospective study, 76 patients with R/M HNSCC treated with pembrolizumab plus 5-FU and either cisplatin (*n* = 41) or carboplatin (*n* = 35) were analysed. The primary endpoint was overall survival (OS); secondary endpoints included progression-free survival (PFS), objective response rate (ORR), and the incidence of grade ≥ 3 haematological and grade ≥ 2 non-haematological adverse events. The median PFS was 4.1 months with cisplatin and 7.1 months with carboplatin (*p* = 0.58). The median OS values were 14.8 and 20.7 months, respectively (*p* = 0.88). The ORR values were similar (34.1% vs. 34.3%, *p* = 0.99). Grade ≥ 3 haematological adverse events were more frequent with cisplatin than with carboplatin (87.8% vs. 48.6%, *p* < 0.01), as were grade ≥ 2 non-haematological adverse events (97.6% vs. 54.3%, *p* < 0.01). Cisplatin did not demonstrate a significant advantage over carboplatin in terms of OS, PFS, and ORR, but was associated with higher toxicity pembrolizumab-based therapy for R/M HNSCC. These findings suggest that carboplatin may represent a reasonable alternative in combination with pembrolizumab.

**Supplementary Information:**

The online version contains supplementary material available at 10.1038/s41598-026-50081-5.

## Introduction

Head and neck squamous cell carcinoma (HNSCC) is the sixth most commonly diagnosed cancer worldwide, with an estimated 891,453 new cases and 458,107 deaths reported in 2020^1^. While survival rates have improved in recent decades, HNSCC remains one of the most fatal cancers globally, highlighting the need for more effective treatment strategies.

Historically, numerous Phase III trials have investigated the use of cisplatin, carboplatin, methotrexate, paclitaxel, 5-fluorouracil (5-FU), and their combination therapies^2–7^. Based on favourable clinical outcomes, cisplatin plus 5-FU was recognized as the standard treatment approach^2–7^. In the EXTREME trial, the addition of cetuximab to a platinum plus 5-FU regimen resulted in a significant improvement in overall survival (OS) for first-line treatment of head and neck cancer, compared with platinum and 5-FU^8^. Owing to the high toxicity of cisplatin and its limited tolerability, the EXTREME trial allowed for the use of carboplatin as a substitute^8,9^. Although based on retrospective analysis, reports from the EXTREME era suggested that cisplatin was associated with a higher objective response rate (ORR), while OS was comparable between cisplatin and carboplatin^10,11^.

Recently, the EXTREME regimen has been replaced by pembrolizumab plus platinum and 5-FU, which have become the new standard of care for the first-line treatment of recurrent or metastatic (R/M) HNSCC^12–14^. Although carboplatin remains an acceptable option, whether it is a more suitable combination partner in the era of immune checkpoint inhibitors (ICIs) remains unclear. Treatment of R/M HNSCC is primarily palliative, focusing on disease control and symptom relief to extend survival and maintain the quality of life^15,16^. To identify the optimal platinum agent for ICI-based regimens, we assessed the efficacy and tolerability of cisplatin and carboplatin. Therefore, this study was designed as an exploratory real-world analysis to evaluate the impact of platinum choice in ICI-based therapy.

## Methods

### Ethics approval and consent to participate

This retrospective study was conducted following the ethical standards outlined in the Declaration of Helsinki and was approved by the Institutional Review Board of the Japanese Foundation for Cancer Research (Approval No. 2025-GB-040). Given the retrospective design of the study, the requirement for written informed consent was waived. Instead, information regarding the study was disclosed on the institutional website, and patients were provided with the opportunity to opt out.

### Study design and patients

This retrospective study was performed at the Cancer Institute Hospital, Japanese Foundation for Cancer Research, and NHO Nagoya Medical Centre between December 2019 and March 2025. Patients with R/M HNSCC originating from the oropharynx, oral cavity, hypopharynx, or larynx were included in the study. The eligibility criteria also included an Eastern Cooperative Oncology Group performance status (ECOG PS) score of 0–2, age ≥ 18 years, and at least one evaluable lesion, as defined by the Response Evaluation Criteria in Solid Tumours (RECIST). All the patients received pembrolizumab combined with cisplatin, carboplatin, and 5-FU. Patients who received systemic platinum-based therapy or developed platinum-refractory disease within 6 months were excluded. The following clinical variables were included in the analysis: age, sex, combined positive score (CPS), creatinine clearance (Ccr), body mass index (BMI), prior exposure to platinum-based chemotherapy, p16 status, ECOG PS, smoking history, and primary tumour site.

### Procedures

Patients received pembrolizumab in combination with either carboplatin (area under the curve [AUC], 5 mg/m²) or cisplatin (100 mg/m²), along with 5-FU (1000 mg/m² per day for 4 consecutive days), administered every 3 weeks for up to 6 cycles. After completion of six cycles, pembrolizumab monotherapy was continued as maintenance treatment. The dose reduction criteria and extent of dose modification for each drug were determined at the discretion of the treating physician. The relative dose intensity (RDI) was defined as the ratio of the actual dose delivered per unit time to the planned dose per unit time. Cumulative relative dose intensity (cRDI) was calculated as the ratio of total actual dose to total planned dose; both metrics were evaluated for the initial chemo-immunotherapy combination phase (cycles 1–6). Treatment discontinuation was defined as failure to complete the six planned chemotherapy cycles.

### Outcomes

The primary endpoint was OS, defined as the time from the initiation of first-line therapy to death from any cause or censoring at the last follow-up. Secondary endpoints included progression-free survival (PFS), defined as the time from treatment initiation to documented disease progression or death from any cause, whichever occurred first. Patients without an event were censored at the last follow-up. Additional secondary points were the incidence of grade ≥ 2 non-haematological adverse events (NHAEs) and grade ≥ 3 haematological adverse events (HAEs), and the ORR. Adverse events were recorded and graded according to the Common Terminology Criteria for Adverse Events (CTCAE) version 5.0. For each adverse event category, the highest grade observed during the entire treatment course was used for analysis. All tumour responses were assessed using the RECIST version 1.1.

### Statistical analysis

All statistical analyses were performed using Stata SE software version 18.0; (StataCorp, College Station, TX, USA). Continuous variables were summarised as medians and compared between groups using the Wilcoxon rank-sum test. Categorical variables were analysed using the chi-squared test or Fisher’s exact test, with the latter applied when the expected cell count was < 5. The median follow-up duration was calculated using the reverse Kaplan–Meier method. Survival curves were estimated using the Kaplan–Meier method and compared using the log-rank test. Univariate and multivariate Cox proportional hazards regression analyses were conducted to identify the prognostic factors. The proportional hazards assumption was assessed using Schoenfeld residuals. In the multivariate analysis, variables that violated this assumption were analysed using stratified Cox regression models. In Cox proportional hazards regression analyses, age, Ccr, and BMI were treated as continuous variables. Statistical significance was set at a two-sided p-value < 0.05.

## Results

Between December 2019 and March 2025, 76 eligible patients with R/M HNSCC were included in the analysis; cisplatin and carboplatin were administered to 41 and 35 patients, respectively. The baseline patient characteristics stratified according to treatment regimen are summarised in Table 1.

**Table 1:** Baseline patient characteristics stratified by treatment group.

	All (*N* = 76)	Cisplatin (*N* = 41)	Carboplatin (*N* = 35)	*p*-value
Age, years, median (range)				< 0.01
	66 (28–78)	57 (28–76)	73 (56–78)	
BMI, kg/m^2^, median (range)				0.997
	21.0 (13.4–30.6)	21.1 (13.4–30.6)	20.5 (15.6–28.7)	
Ccr, mL/min, median (range)				**< 0.01**
	81.0 (39.8–177.9)	97.4 (61.1–177.9)	60.0 (39.8–104.2)	
Sex, n (%)				0.60
	Male: 61 (80.3)	Male: 32 (78.0)	Male: 29 (82.9)	
	Female: 15 (19.7)	Female: 9 (22.0)	Female: 6 (17.1)	
p16 status, n (%)				0.27
	Negative: 69 (92.0)	Negative: 39 (95.1)	Negative: 30 (88.2)	
	Positive: 6 (8.0)	Positive: 2 (4.9)	Positive: 4 (11.8)	
	Missing: 1 (1.3)	Missing: 0 (0.0)	Missing: 1 (2.9)	
CPS status, n (%)				**0.049**
	1 ≤ CPS < 20: 33 (43.4)	1 ≤ CPS < 20: 13 (31.7)	1 ≤ CPS < 20: 20 (57.1)	
	CPS ≥ 20: 40 (52.6)	CPS ≥ 20: 25 (61.0)	CPS ≥ 20: 15 (42.9)	
	Missing: 3 (3.9)	Missing: 3 (7.3)	Missing: 0 (0.0)	
Smoking history, n (%)				**0.04**
	Smoker: 54 (71.1)	Smoker: 25 (61.0)	Smoker: 29 (82.9)	
	Never: 22 (28.9)	Never: 16 (39.0)	Never: 6 (17.1)	
Primary tumour site, n (%)				**< 0.01**
	Oral cavity: 34 (44.7)	Oral cavity: 26 (63.4)	Oral cavity: 8 (22.9)	
	Oropharynx: 19 (25.0)	Oropharynx: 7 (17.1)	Oropharynx: 12 (34.3)	
	Hypopharynx: 18 (23.7)	Hypopharynx: 6 (14.6)	Hypopharynx: 12 (34.3)	
	Larynx: 5 (6.6)	Larynx: 2 (4.9)	Larynx: 3 (8.6)	
Diagnosis status, n (%)				0.54
	De novo: 9 (11.8%)	De novo: 4 (9.8)	De novo: 5 (14.3)	
	Recurrent: 67 (88.2%)	Recurrent: 37 (90.2)	Recurrent: 30 (85.7)	
Performance status, n (%)				0.83
	PS0: 27 (35.5%)	PS0: 17 (41.5)	PS0: 10 (28.6)	
	PS1: 40 (52.6%)	PS1: 20 (48.8)	PS1: 20 (57.1)	
	PS2: 9 (11.8%)	PS2: 4 (9.8)	PS2: 5 (14.3)	
Prior platinum therapy, n (%)				0.14
	Present: 26 (34.2%)	Present: 11 (26.8)	Present: 15 (42.9)	
	Absent: 50 (65.8%)	Absent: 30 (73.2)	Absent: 20 (57.1)	

Significant values are in bold.

Data were presented as medians (ranges) for continuous variables and numbers (percentages) for categorical variables. Percentages are calculated excluding cases with missing data. Group comparisons were performed using the Wilcoxon rank-sum test for continuous variables and the chi-square test for categorical variables.

Patients in the cisplatin group were significantly younger (median age: 57 vs. 73 years, *p* < 0.01) and had a higher creatinine clearance (median: 97.4 vs. 60.0%/min, *p* < 0.01) than those in the carboplatin group. The distributions of CPS status (*p* = 0.049), smoking history (*p* = 0.04), and primary tumour site (*p* < 0.01) also differed between the groups.

The treatment delivery metrics are presented in Table 2.

**Table 2:** Treatment delivery characteristics, reasons for discontinuation, and second-/third-line therapy after disease progression by treatment group. Discontinuation reasons are shown as % of all patients in each treatment group, not % of those who discontinued. Post-progression analyses include patients with first progression (N=57). P values were calculated using the Wilcoxon rank-sum test for continuous variables and the chi-squared test or Fisher’s exact test for categorical variables, as appropriate.

	All (N=76)	Cisplatin (N=41)	Carboplatin (N=35)	p-value
Median total RDI, % (range)	79.5 (8.2–100)	75.5 (8.2–100)	81.1 (31.3–100)	0.41
Median 5-FU RDI, % (range)	78.4 (3.3–100)	79.1 (3.3–100)	77.8 (12.8–100)	0.75
Median platinum RDI, % (range)	80.0 (13.0–100)	75.5 (13.0–80.8)	81.5 (29.0–100)	0.16
Median total cRDI, % (range)	62.5 (10.4–100)	53.3 (10.4–100)	66.7 (10.4–84.7)	0.07
Median 5-FU cRDI, % (range)	66.7 (4.2–100)	53.3 (4.2–100)	66.7 (4.2–86.0)	0.23
Median platinum cRDI, % (range)	56.7 (13.3–100)	53.3 (13.3–100)	66.7 (16.7–84.7)	0.02
Completion, n (%)	24 (31.6)	10 (24.4)	14 (40.0)	0.15
Discontinuation, n (%)	52 (68.4)	31 (75.6)	21 (60.0)	0.15
Disease progression	26 (34.2)	14 (34.1)	12 (34.3)	0.99
Adverse events	20 (26.3)	15 (36.6)	5 (14.3)	0.03
Others	6 (7.9)	2 (4.9)	4 (11.4)	0.41

After disease progression	All (N=57)	Cisplatin (N=31)	Carboplatin (N=26)	p-value
Second-line treatment rate, n (%)	30 (52.6)	14 (45.2)	16 (61.5)	0.22
Paclitaxel plus Cetuximab	23 (40.4)	11 (35.5)	12 (46.2)	
Paclitaxel alone	2 (3.5)	2 (6.5)	0 (0.0)	
Tegafur/gimeracil/oteracil potassium	1 (1.8)	0 (0.0)	1 (3.8)	
Others	4 (7.0)	1 (3.2)	3 (11.5)	
Third-line treatment rate, n (%)	8 (14.0)	3 (9.7)	5 (19.2)	0.45
Paclitaxel plus Cetuximab	1 (1.8)	0 (0.0)	1 (3.8)	
Paclitaxel alone	1 (1.8)	0 (0.0)	1 (3.8)	
Tegafur/gimeracil/oteracil potassium	3 (5.3)	1 (3.2)	2 (7.7)	
Others	3 (5.3)	2 (6.5)	1 (3.8)	

Platinum cRDI was significantly lower in the cisplatin group than in the carboplatin group (*p* = 0.02). The proportion of patients who completed all six planned cycles was 24.4% in the cisplatin group and 40.0% in the carboplatin group (*p* = 0.15). To provide additional context regarding the potential impact of treatment completion, exploratory landmark analyses at 4.5 months (corresponding to completion of six cycles) were performed (Supplementary Fig. 1 A, B). Median OS from the landmark was 24.8 months in the completion group and 12.2 months in the AE-related discontinuation group (*p* = 0.12), while median PFS was 12.8 and 17.3 months, respectively (*p* = 0.27). Treatment discontinuation owing to adverse events was more frequently observed in the cisplatin group than in the carboplatin group (36.6% vs. 14.3%; *p* = 0.03). The median follow-up period was 32.4 months. The ORR was 34.2%, with comparable rates in the cisplatin (34.1%) and carboplatin (34.3%) groups.

Detailed outcome data are shown in Table 3.

**Table 3:** Clinical outcomes and tumour response.

	All (*N* = 76)	Cisplatin (*N* = 41)	Carboplatin (*N* = 35)	*p*-value
Median follow-up period, months (range)	32.4 (0.4–62.3)	34.7 (1.3–62.3)	25.4 (0.4–57.5)	
Median PFS, months (95% CI)	5.2 (3.9–7.8)	4.1 (3.3–7.8)	7.1 (4.2–9.0)	0.68
Median OS, months (95% CI)	19.9 (13.8–27.6)	14.8 (8.1–28.2)	20.7 (15.0–29.3)	0.88
ORR, n (%)	26 (34.2)	14 (34.1)	12 (34.3)	0.99
Complete response, n (%)	10 (13.2)	7 (17.1)	3 (8.6)	
Partial response, n (%)	16 (21.1)	7 (17.1)	9 (25.7)	
Stable disease, n (%)	28 (36.8)	15 (36.6)	13 (37.1)	
Progressive disease, n (%)	22 (29.0)	12 (29.3)	10 (28.6)	

Follow-up duration, PFS, and OS are presented as median values with corresponding ranges or 95% CI. ORR comprised complete and partial responses per RECIST v1.1. Group comparisons were performed using the chi-squared test for ORR and log-rank test for PFS, and OS.

**Fig. 1:**
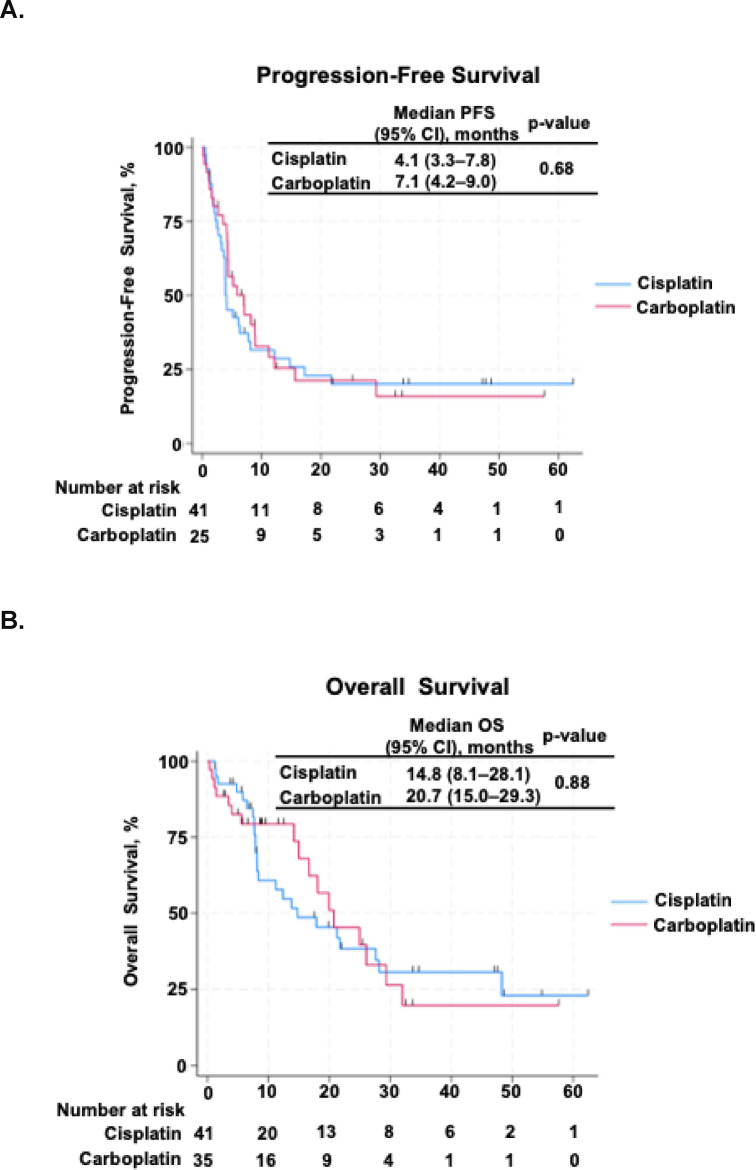
Kaplan–Meier curves for progression-free survival (**A**) and overall survival (**B**) stratified by platinum agent.

Median PFS was 4.1 months in the cisplatin group and 7.1 months in the carboplatin group (Fig. 1A). Median OS was 14.8 and 20.7 months, respectively (Fig. 1B).

In the univariate analysis, the HR for PFS with carboplatin compared to cisplatin was 0.86 (95% CI: 0.51–1.46, *p* = 0.58), and the HR for OS was 0.95 (95% CI: 0.51–1.78, *p* = 0.88). PS 1 and PS 2 were strongly associated with shorter PFS (*p* = 0.04 and *p* = 0.01), and OS (*p* < 0.01 and *p* = 0.01, respectively). CPS ≥ 20 was significantly associated with longer PFS (*p* = 0.03). Stratified analyses according to CPS (≥ 20 vs. 1–19) showed no significant differences in OS or PFS between cisplatin and carboplatin within each subgroup (Supplementary Fig. 2A–D). These variables were then included as covariates in the multivariate model. In the multivariate analysis, the HRs for carboplatin were 0.70 (95% CI: 0.40–1.23) for PFS and 0.68 (95% CI: 0.34–1.34) for OS. The results of the univariable and multivariable analyses are summarised in Table 4.

**Table 4:** Univariate and multivariate Cox regression analyses for PFS and OS.

Variable	PFS univariate analysis			OS univariate analysis		
	Hazard Ratio	95%CI	*p*-value	Hazard Ratio	95%CI	*p*-value
Age	0.99	0.97–1.01	0.40	1.01	0.98–1.04	0.57
BMI	0.95	0.87–1.04	0.29	0.92	0.84–1.01	0.09
Ccr	1	0.99–1.01	0.98	0.99	0.98–1.00	0.22
Sex						
Female	1			1		
Male	1.26	0.63–2.50	0.50	1.54	0.71–3.37	0.26
Regimen						
Cisplatin	1			1		
Carboplatin	0.86	0.51–1.46	0.58	0.95	0.51–1.78	0.88
p16 status						
Negative	1			1		
Positive	1.39	0.59–3.26	0.47	0.86	0.31–2.42	0.77
CPS status						
1 ≦ CPS < 20	1			1		
CPS ≧ 20	0.54	0.31–0.93	**0.03**	0.62	0.33–1.16	0.14
Smoking history						
Non-Smoker	1			1		
Smoker	1.13	0.62–2.01	0.68	1.22	0.61–2.44	0.58
Primary tumour site						
Oropharynx	1			1		
Hypopharynx	0.58	0.28–1.24	0.62	0.88	0.36–2.13	0.78
Larynx	0.41	0.12–1.43	0.43	0.58	0.16–2.1	0.40
Oral cavity	0.66	0.35–1.25	0.65	0.78	0.37–1.69	0.54
Diagnosis status						
De novo	1			1		
Recurrent	0.91	0.41–2.02	0.82	1.16	0.41–3.29	0.78
Performance status						
0	1			1		
1	1.85	1.03–3.32	**0.04**	2.98	1.44–6.19	**< 0.01**
2	2.93	1.26–6.79	**0.01**	4.13	1.39–12.28	**0.01**
Prior platinum therapy						
Absent	1			1		
Present	1.24	0.73–2.13	0.43	1.56	0.84–2.88	0.16

Variable	PFS multivariate analysis			OS multivariate analysis		
	Hazard Ratio	95%CI	*p*-value	Hazard Ratio	95%CI	*p*-value
Regimen						
Cisplatin	1			1		
Carboplatin	0.70	0.40–1.23	0.22	0.68	0.34–1.34	0.27
CPS status						
1 ≦ CPS < 20	1			1		
CPS ≧ 20	0.44	0.25–0.78	**< 0.01**	0.54	0.27–1.07	0.07
Performance status						
0	1			1		
1	2.07	1.13–3.80	**0.02**	2.84	1.35–5.98	**< 0.01**
2	3.53	1.48–8.41	**< 0.01**	4.29	1.42–12.89	**0.01**

Significant values are in bold.

Hazard ratios (HRs), 95% confidence intervals (CIs), and p values were calculated for each clinical variable. Variables with *p* < 0.05 in univariate analyses for either PFS or OS were included in the corresponding multivariate models. Reference categories are indicated for the categorical variables.

Grade ≥ 3 HAEs were significantly more common in the cisplatin group than in the carboplatin group (87.8% vs. 48.6%, *p* < 0.01). Specifically, decreased white blood cell count (46.3% vs. 17.1%, *p* < 0.01), decreased neutrophil count (65.9% vs. 34.3%, *p* < 0.01), and febrile neutropenia (17.1% vs. 0%, *p* = 0.01) were relatively frequent in the cisplatin group. Grade ≥ 2 NHAEs were also more frequently observed in the cisplatin (97.6% vs. 54.3%, *p* < 0.01). Notably, nausea (53.7% vs. 11.4%, *p* < 0.01), anorexia (46.3% vs. 22.9%, *p* = 0.03), malaise (22.0% vs. 2.9%, *p* = 0.02), and diarrhoea (24.4% vs. 2.9%, *p* < 0.01) occurred relatively often in patients receiving cisplatin. A summary of adverse events is provided in Table 5.

**Table 5:** Summary of treatment-related adverse events.

	All (*N* = 76)	Cisplatin (*N* = 41)	Carboplatin (*N* = 35)	*p*-value
G3 ≥ Haematological AEs, *n* (%)	53 (69.7)	36 (87.8)	17 (48.6)	< 0.01
White blood cell decreased, n (%)	25 (32.9)	19 (46.3)	6 (17.1)	**< 0.01**
Neutrophil count decreased, n (%)	39 (51.3)	27 (65.9)	12 (34.3)	**< 0.01**
Febrile neutropenia, n (%)	7 (9.2)	7 (17.1)	0 (0.0)	**0.01**
Platelet count decreased, n (%)	5 (6.6)	2 (4.9)	3 (8.6)	0.66
Anemia, n (%)	16 (21.1)	10 (24.4)	6 (17.1)	0.59
G2 ≥ Non-Haematological AEs, n (%)	59 (77.6)	40 (97.6)	19 (54.3)	**< 0.01**
Nausea, n (%)	26 (34.2)	22 (53.7)	4 (11.4)	**< 0.01**
Anorexia, n (%)	27 (35.5)	19 (46.3)	8 (22.9)	**0.03**
Malaise, n (%)	10 (13.2)	9 (22.0)	1 (2.9)	**0.02**
Mucositis oral, n (%)	11 (14.5)	7 (17.1)	4 (11.4)	0.49
Diarrhoea, n (%)	11 (14.5)	10 (24.4)	1 (2.9)	**< 0.01**
Constipation, n (%)	12 (15.8)	6 (14.6)	6 (17.1)	0.77
Creatinine increased, n (%)	4 (5.3)	4 (9.8)	0 (0.0)	0.12
ALT increased, n (%)	5 (6.6)	3 (7.3)	2 (5.7)	1.00
AST increased, n (%)	3 (3.9)	2 (4.9)	1 (2.9)	1.00
GGT increased, n (%)	3 (3.9)	1 (2.4)	2 (5.7)	0.59
ALP increased, n (%)	1 (1.3)	0 (0.0)	1 (2.9)	0.46
Hyponatremia, n (%)	6 (7.9)	4 (9.8)	2 (5.7)	0.68
Hypokalemia, n (%)	2 (2.6)	2 (4.9)	0 (0.0)	0.50
Hyperkalemia, n (%)	1 (1.3)	0 (0.0)	1 (2.9)	0.46
Hypocalcemia, n (%)	1 (1.3)	1 (2.4)	0 (0.0)	1.00
Hypomagnesemia, n (%)	1 (1.3)	1 (2.4)	0 (0.0)	1.00
Eczema, n (%)	5 (6.6)	3 (7.3)	2 (5.7)	1.00
Hearing impaired, n (%)	3 (3.9)	3 (7.3)	0 (0.0)	0.25
Peripheral sensory neuropathy, n (%)	2 (2.6)	2 (4.9)	0 (0.0)	0.50
Thromboembolic event, n (%)	1 (1.3)	1 (2.4)	0 (0.0)	1.00
Pneumonitis, n (%)	1 (1.3)	1 (2.4)	0 (0.0)	1.00
Adrenal insufficiency, n (%)	4 (5.3)	3 (7.3)	1 (2.9)	0.62
Dizziness, n (%)	1 (1.3)	0 (0.0)	1 (2.9)	0.46
Dysgeusia, n (%)	2 (2.6)	2 (4.9)	0 (0.0)	0.50

Significant values are in bold.

Grade ≥ 3 haematological and Grade ≥ 2 non-haematological adverse events were evaluated according to CTCAE version 5.0. Comparisons between the cisplatin and carboplatin groups were conducted using the chi-squared test or Fisher’s exact test.

## Discussion

Cisplatin has long been a cornerstone in the treatment of R/M HNSCC owing to its potent antitumour activity^2–7^. It remains preferentially used in patients with a good PS and preserved organ function, even in the post-EXTREME era^8^. However, the substantial toxicity of cisplatin can adversely affect health-related quality of life^9^. In clinical practice, carboplatin is frequently selected for patients who are older or have impaired renal function, as was observed in our study. Consistent with this clinical practice, a real-world retrospective study of the EXTREME regimen conducted in Japan reported that carboplatin-based treatment was associated with fewer adverse event-related discontinuations^10^.

In our retrospective analysis based on real-world data, cisplatin demonstrated no superiority over carboplatin in terms of OS and PFS. Moreover, it failed to show any advantage in ORR when used in combination with pembrolizumab and 5-FU. Despite the inclusion of patients with an ECOG PS 2, the observed median PFS was consistent with that reported in the KEYNOTE-048 trial, and the median OS showed a trend toward improvement^12,13^. These OS outcomes were also similar to those observed in the Japanese subgroup of KEYNOTE-048^17,18^.

Although CPS was not significantly associated with OS in the univariate analysis, it was retained in the multivariate model because of its significant association with PFS and its established role as a prognostic biomarker in R/M HNSCC^12,13^. To evaluate the robustness of our findings, we conducted multiple sensitivity analyses, including models incorporating additional clinical covariates (p16 status, primary tumour site, smoking history, age, and Ccr), as well as truncated Cox models at 24 months. Because of the limited sample size and number of events, we adopted a parsimonious modeling strategy and avoided over-parameterization in the primary model to minimize the risk of overfitting^19^. All sensitivity analyses of OS and PFS demonstrated results consistent with those of the primary analysis (Supplementary Table 1 A, B).

Grade ≥ 2 non-haematologic toxicities were analysed, as they are considered clinically meaningful events that compromise quality of life, and affect treatment decision. The incidence of both grade ≥ 3 HAEs and grade ≥ 2 NHAEs was significantly higher in the cisplatin group than in the carboplatin group. Grade ≥ 3 NHAEs were also more frequently observed in the cisplatin group (Supplementary Table 2 A). In contrast, the number of grade ≥ 2 immune-related adverse events was comparable between the two groups (Supplementary Table 2B). Notably, platinum cRDI was significantly lower in the cisplatin group, and the proportion of AE-related discontinuations was higher than that in the carboplatin group. Considering its increased toxicity and decreased treatment delivery, carboplatin may be a reasonable option, particularly when sustained platinum administration is prioritised. Compared to the overall population in KEYNOTE-048, the prevalence of severe adverse events in our cohort was high. However, the toxicity profile was similar to that reported in a Japanese subgroup, suggesting that ethnic and clinical practice factors may influence the differences^17,18^. The regimen of cisplatin at 100 mg/m² on day 1 combined with continuous intravenous infusion of 5-FU at 1,000 mg/m² per day from days 1 to 4 has been reported to be excessively intensive in Japanese patients^20^. In our cohort, only 5 of 42 patients in the cisplatin group were initiated on a dose-attenuated regimen (cisplatin 80 mg/m² on day 1 combined with 5-FU 800 mg/m² per day on days 1 to 4), indicating that the majority received the full-dose regimen. Therefore, the high incidence of adverse events observed in this study may be attributable to this dosing intensity. Given the limited number of patients treated with a dose-attenuated regimen, a direct comparison between dose-attenuated cisplatin and carboplatin was not feasible. These findings suggest that the observed differences in toxicity between cisplatin and carboplatin may partly reflect treatment intensity rather than intrinsic differences between the agents. Although fluoropyrimidine-related toxicity may contribute to adverse events, the relative dose intensity of 5-FU was comparable between groups. Clinically relevant *DPYD* variants are rare in East Asian populations, suggesting that fluoropyrimidine exposure is unlikely to have been a major driver of the observed differences^21^. Adverse event profiles in R/M HNSCC are known to vary across studies, and a prior analysis also highlighted variability in toxicity outcomes, suggesting that factors beyond platinum selection may influence adverse events^22^.

In the era of ICIs, their potent antitumour activity appears to diminish the clinical relevance of the selection between cisplatin and carboplatin. Given its favourable toxicity profile, carboplatin may be considered an alternative. While our study focused on platinum selection, chemotherapy choice in ICI-based regimens is increasingly guided by the consideration of toxicity^23,24^. Future research is required to clarify the optimal chemotherapy backbone in ICI-based regimens and refine treatment approaches that balance efficacy and tolerability.

### Limitations

First, the retrospective design may have introduced selection bias and residual confounding. Although we adjusted for key prognostic variables in multivariable and sensitivity analyses, unmeasured confounders cannot be excluded.

Second, the sample size was relatively small, which may have limited statistical power.

Third, the median follow-up duration differed between treatment groups, which may have influenced OS estimates; however, truncated analyses at 24 months yielded results consistent with the primary analysis.

Finally, as most patients were Japanese, the applicability of our findings to other populations may be limited.

In this context, this study should be interpreted as an exploratory, hypothesis-generating analysis rather than a confirmatory comparison between platinum agents, and the findings should be validated in prospective studies.

## Conclusion

In conclusion, cisplatin did not demonstrate a significant advantage over carboplatin in terms of OS, PFS, and ORR in this retrospective study. Additionally, cisplatin was associated with higher toxicity in the context of ICI-based therapy for R/M HNSCC. These findings suggest that carboplatin may represent a reasonable alternative in combination with pembrolizumab when considering the balance between efficacy and safety. Further investigation is necessary to validate these findings and inform future treatment strategies.

## Supplementary Information

Below is the link to the electronic supplementary material.

Supplementary material 1

## Data Availability

The data supporting the findings of this study are not publicly available owing to patient confidentiality but can be obtained from the corresponding author upon reasonable request.

## References

[1] Bray, F. et al. Global cancer statistics 2022: GLOBOCAN estimates of incidence and mortality worldwide for 36 cancers in 185 countries. *CA Cancer J. Clin.* **74**, 229–263 (2024). 38572751 10.3322/caac.21834

[2] Forastiere, A. A. et al. Randomized comparison of cisplatin plus fluorouracil and carboplatin plus fluorouracil versus methotrexate in advanced squamous-cell carcinoma of the head and neck: A Southwest Oncology Group study. *J. Clin. Oncol.* **10**, 1245–1251 (1992). 1634913 10.1200/JCO.1992.10.8.1245

[3] Jacobs, C. et al. A phase III randomized study comparing cisplatin and fluorouracil as single agents and in combination for advanced squamous cell carcinoma of the head and neck. *J. Clin. Oncol.* **10**, 257–263 (1992). 1732427 10.1200/JCO.1992.10.2.257

[4] Schornagel, J. H. et al. Randomized phase III trial of edatrexate versus methotrexate in patients with metastatic and/or recurrent squamous cell carcinoma of the head and neck: A European Organization for Research and Treatment of Cancer Head and Neck Cancer Cooperative Group study. *J. Clin. Oncol.* **13**, 1649–1655 (1995). 7602354 10.1200/JCO.1995.13.7.1649

[5] Schrijvers, D. et al. Phase III trial of modulation of cisplatin/fluorouracil chemotherapy by interferon alfa-2b in patients with recurrent or metastatic head and neck cancer. Head and Neck Interferon Cooperative Study Group. *J. Clin. Oncol.* **16**, 1054–1059 (1998). 9508190 10.1200/JCO.1998.16.3.1054

[6] Forastiere, A. A. et al. Phase III comparison of high-dose paclitaxel + cisplatin + granulocyte colony-stimulating factor versus low-dose paclitaxel + cisplatin in advanced head and neck cancer: Eastern Cooperative Oncology Group Study E1393. *J. Clin. Oncol.* **19**, 1088–1095 (2001). 11181673 10.1200/JCO.2001.19.4.1088

[7] Gibson, M. K. et al. Randomized phase III evaluation of cisplatin plus fluorouracil versus cisplatin plus paclitaxel in advanced head and neck cancer (E1395): An intergroup trial of the Eastern Cooperative Oncology Group. *J. Clin. Oncol.* **23**, 3562–3567 (2005). 15908667 10.1200/JCO.2005.01.057

[8] Vermorken, J. B. et al. Platinum-based chemotherapy plus cetuximab in head and neck cancer. *N Engl. J. Med.* **359**, 1116–1127 (2008). 18784101 10.1056/NEJMoa0802656

[9] Lokich, J. & Anderson, N. Carboplatin versus cisplatin in solid tumors: An analysis of the literature. *Ann. Oncol.* **9**, 13–21 (1998). 9541678 10.1023/a:1008215213739

[10] Sano, D. et al. Real-world treatment outcomes of the EXTREME regimen as first-line therapy for recurrent/metastatic squamous cell carcinoma of the head and neck: A multi-center retrospective cohort study in Japan. *Anticancer Res.* **39**, 6819–6827 (2019). 31810948 10.21873/anticanres.13898

[11] Fukuda, N. et al. Comparison of the efficacy and safety of the EXTREME regimen for treating recurrent or metastatic head and neck squamous cell carcinoma in older and younger adult patients. *Cancer Rep. 4 Cancer Rep. (Hoboken)*. **4**, e1322 (2021). 33295110 10.1002/cnr2.1322 PMC8451378

[12] Burtness, B. et al. Pembrolizumab alone or with chemotherapy versus cetuximab with chemotherapy for recurrent or metastatic squamous cell carcinoma of the head and neck (KEYNOTE-048): A randomised, open-label, phase 3 study. *Lancet* **394**, 1915–1928 (2019). 31679945 10.1016/S0140-6736(19)32591-7

[13] Burtness, B. et al. Pembrolizumab alone or with chemotherapy for recurrent/metastatic head and neck squamous cell carcinoma in KEYNOTE-048: Subgroup analysis by programmed death ligand-1 combined positive score. *J. Clin. Oncol.* **40**, 2321–2332 (2022). 35333599 10.1200/JCO.21.02198 PMC9287281

[14] Harrington, K. J. et al. Pembrolizumab with or without chemotherapy in recurrent or metastatic head and neck squamous cell carcinoma: Updated results of the phase III KEYNOTE-048 study. *J. Clin. Oncol.* **41**, 790–802 (2023). 36219809 10.1200/JCO.21.02508 PMC9902012

[15] Rischin, D. et al. Pembrolizumab alone or with chemotherapy for recurrent or metastatic head and neck squamous cell carcinoma: Health-related quality-of-life results from KEYNOTE-048. *Oral Oncol.* **128**, 105815 (2022). 35381576 10.1016/j.oraloncology.2022.105815

[16] Peyrade, F. et al. Systemic treatment and medical management of metastatic squamous cell carcinoma of the head and neck: Review of the literature and proposal for management changes. *Oral Oncol.* **49**, 482–491 (2013). 23415727 10.1016/j.oraloncology.2013.01.005

[17] Takahashi, S. et al. First-line pembrolizumab ± chemotherapy for recurrent/metastatic head and neck cancer: Japanese subgroup of KEYNOTE-048. *Int. J. Clin. Oncol.* **27**, 1805–1817 (2022). 36264378 10.1007/s10147-022-02233-6 PMC9700657

[18] Oridate, N. et al. First-line pembrolizumab with or without chemotherapy for recurrent or metastatic head and neck squamous cell carcinoma: 5-year follow-up of the Japanese population of KEYNOTE–048. *Int. J. Clin. Oncol.* **29**, 1825–1839 (2024). 39382718 10.1007/s10147-024-02632-x PMC11588814

[19] Babyak, M. A. What you see may not be what you get: A brief, nontechnical introduction to overfitting in regression-type models. *Psychosom. Med.* **66**, 411–421 (2004). 15184705 10.1097/01.psy.0000127692.23278.a9

[20] Kiyota, N. et al. Systemic chemotherapy with cisplatin plus 5-FU (PF) for recurrent or metastatic squamous cell carcinoma of the head and neck (R/M SCCHN): Efficacy and safety of a lower dose of PF (80/800) at a single institution in Japan. *Jpn J. Clin. Oncol.* **39**, 225–230 (2009). 19211574 10.1093/jjco/hyp002

[21] Hishinuma, E. et al. Functional characterization of 21 allelic variants of dihydropyrimidine dehydrogenase identified in 1070 Japanese individuals. *Drug Metab. Dispos.* **46**, 1083–1090 (2018). 29769267 10.1124/dmd.118.081737

[22] Soulières, D. et al. Cetuximab plus platinum-based chemotherapy in head and neck squamous cell carcinoma: A randomized, double-blind safety study comparing cetuximab produced from two manufacturing processes using the EXTREME study regimen. *BMC Cancer*. **16**, 19 (2016). 26768732 10.1186/s12885-016-2064-0 PMC4714495

[23] Dzienis, M. et al. Pembrolizumab plus carboplatin and paclitaxel as first-line therapy for recurrent/metastatic head and neck squamous cell carcinoma (KEYNOTE-B10): A single-arm phase IV trial. *J. Clin. Oncol.* **42**, 2989–2999 (2024). 39038265 10.1200/JCO.23.02625 PMC11361359

[24] Sun, L. et al. Platinum/taxane/pembrolizumab vs platinum/5FU/pembrolizumab in recurrent/metastatic head and neck squamous cell carcinoma (r/m HNSCC). *Oral Oncol.* **158**, 106997 (2024). 39159526 10.1016/j.oraloncology.2024.106997

