# Supplementary material for: Real-world platinum selection in immune checkpoint inhibitor-based therapy for recurrent or metastatic head and neck cancer: an exploratory multi-institutional retrospective study
Source: Sci Rep. 2026 Apr 24;16:18876. doi: 10.1038/s41598-026-50081-5 (PMC13276374; doi:10.1038/s41598-026-50081-5)
Supplement: Supplementary file 1 — Supplementary material 1 [file 41598_2026_50081_MOESM1_ESM.docx]

**A.**

**
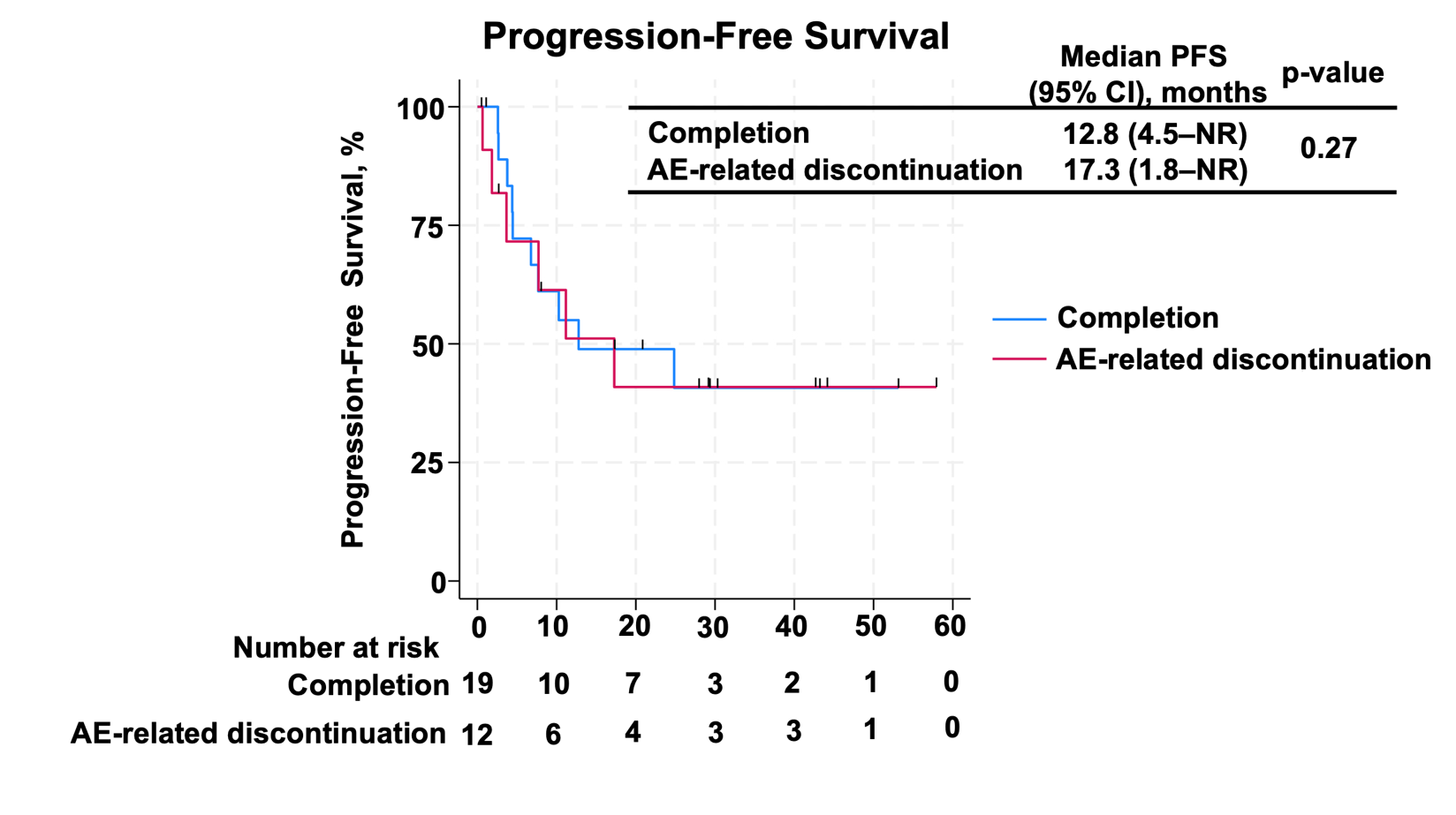
**

**B.**

**
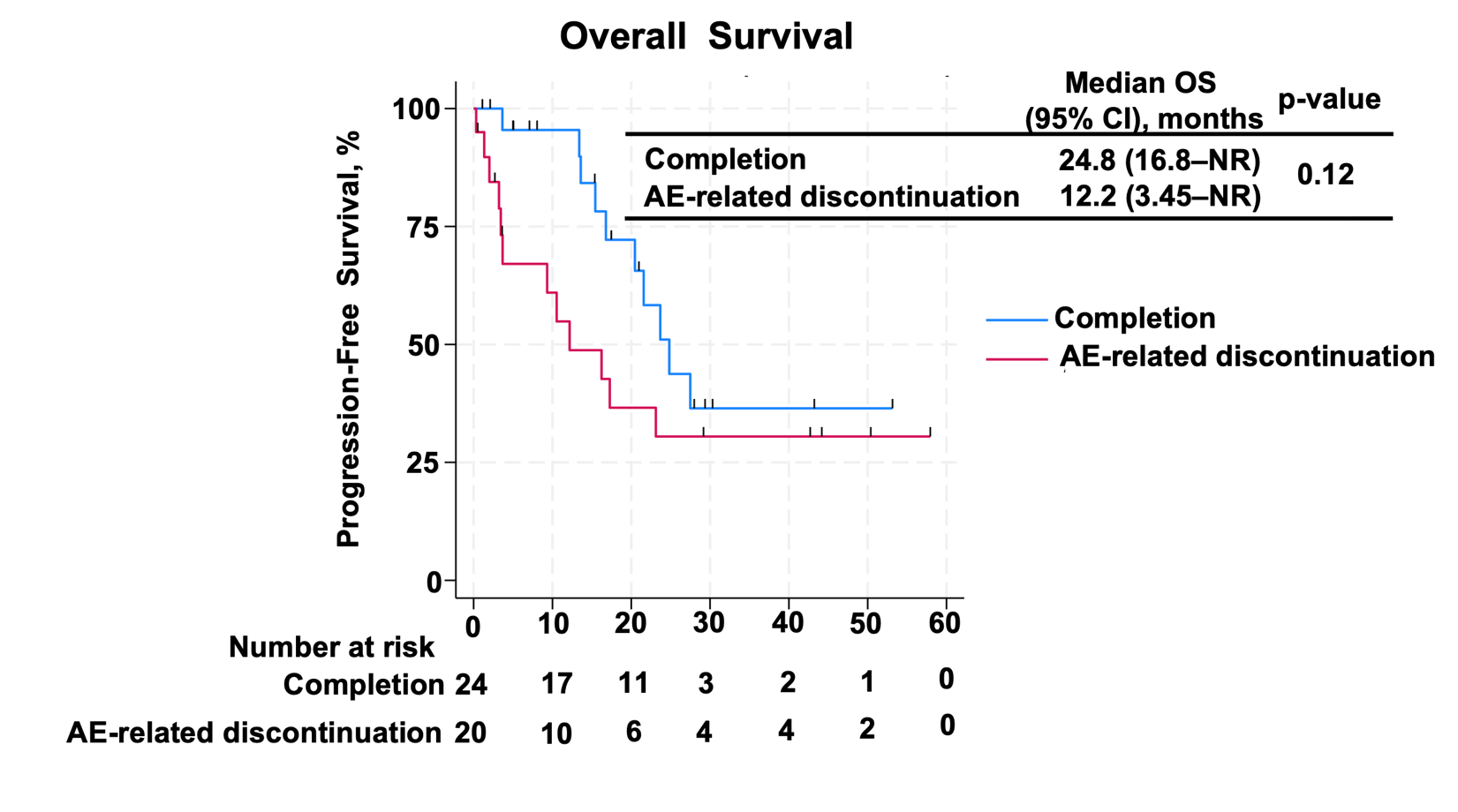
**

**Supplementary Figure 1.**

**Kaplan–Meier curves of progression-free survival (PFS) and overall survival (OS) according to treatment status.** (A) PFS; (B) OS. Patients were categorized into treatment completion and adverse event (AE)-related discontinuation. Median survival with 95% confidence intervals (CIs) and log-rank p-values are shown. Tick marks indicate censored observations; numbers at risk are provided below.

**A.**

**
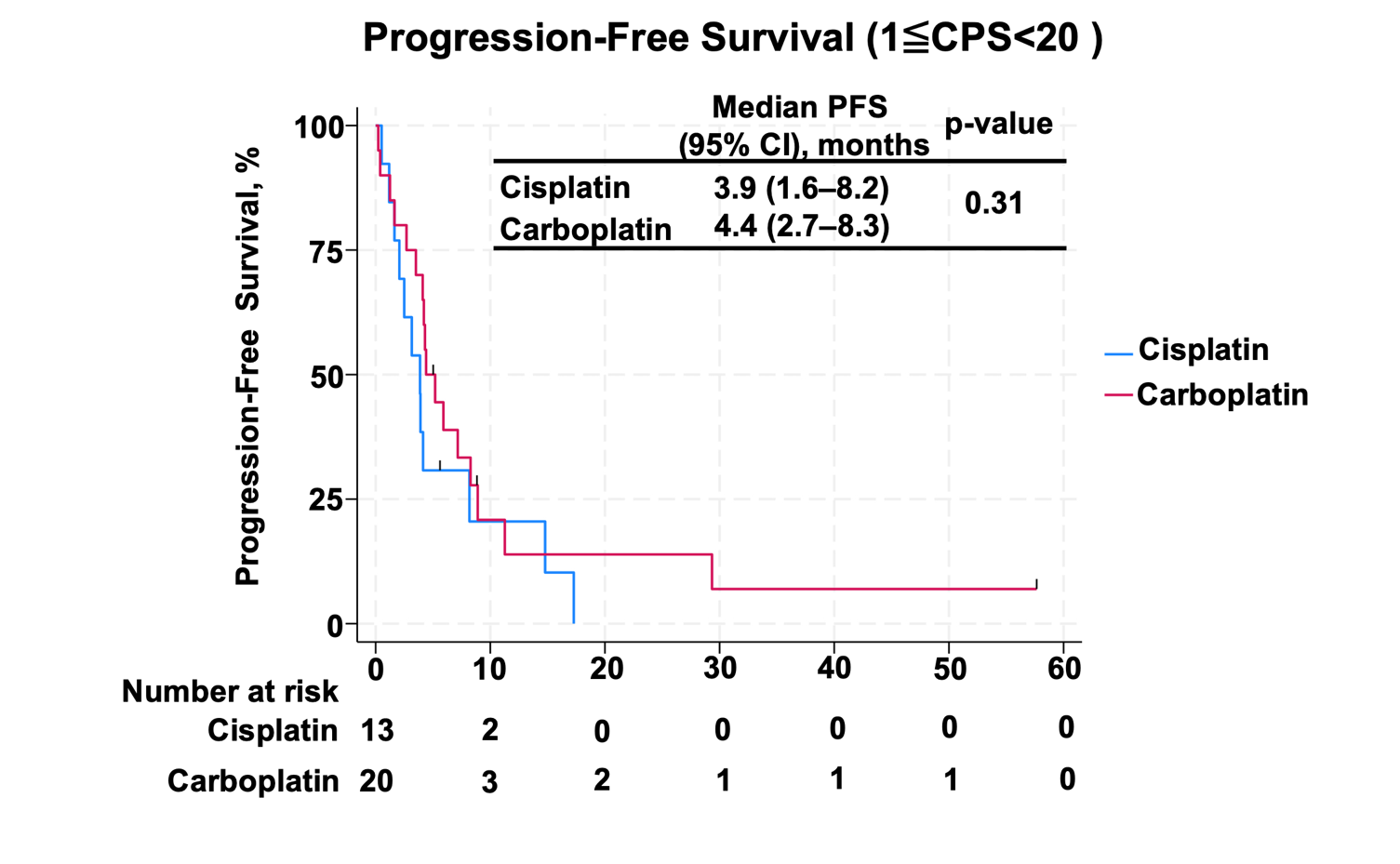
**

**B.**

**
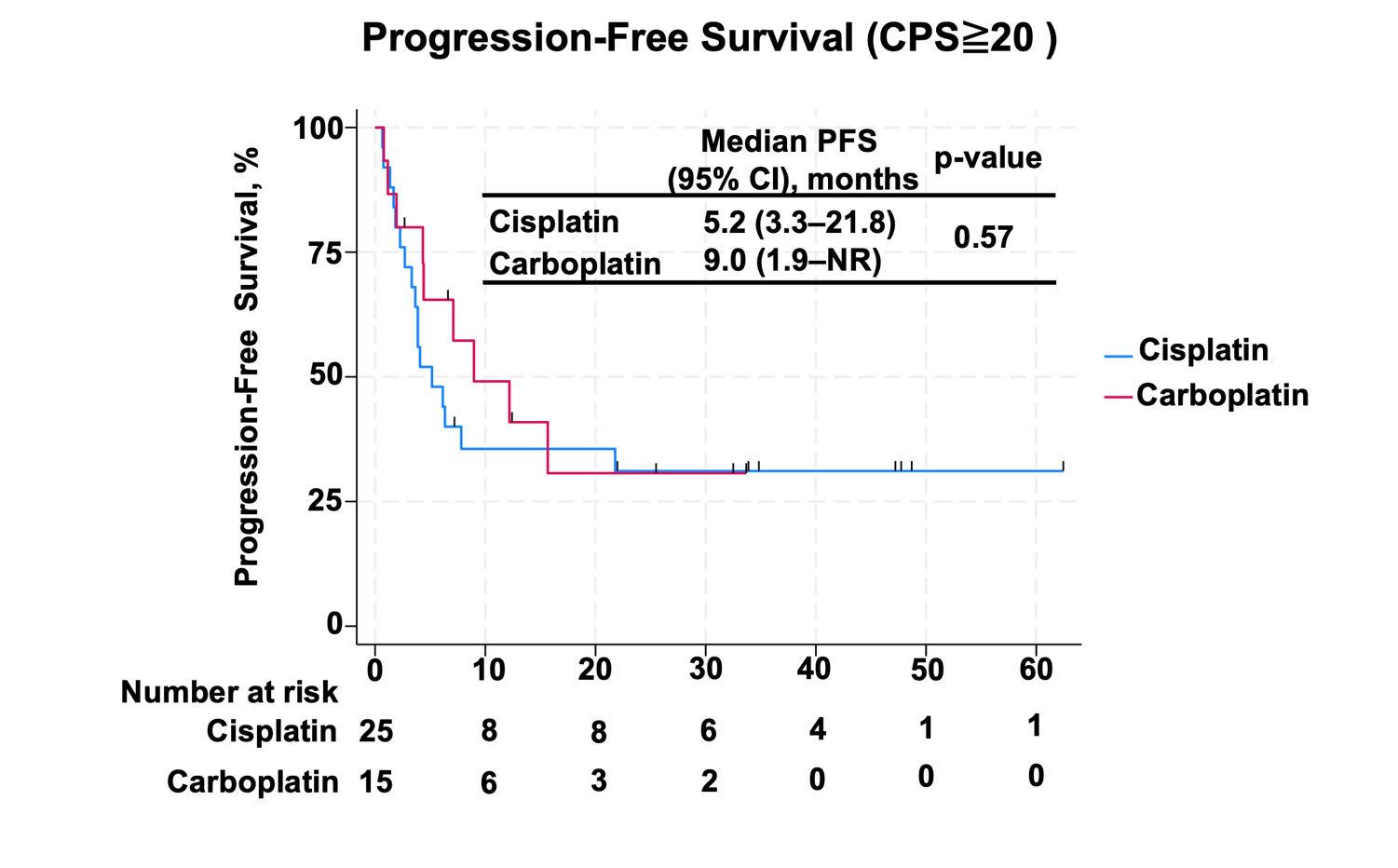
**

**C.**

**
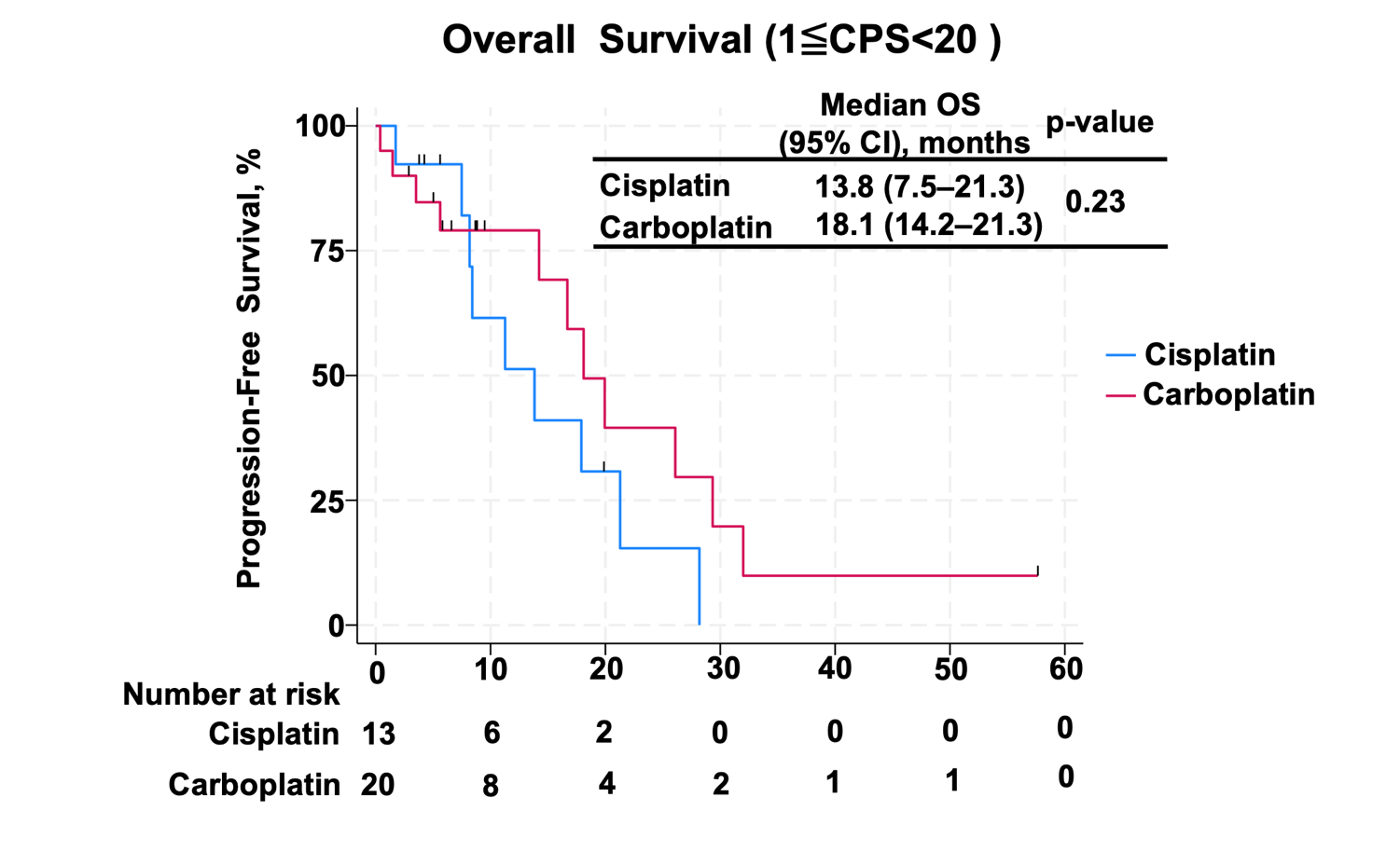
**

**D.**

**
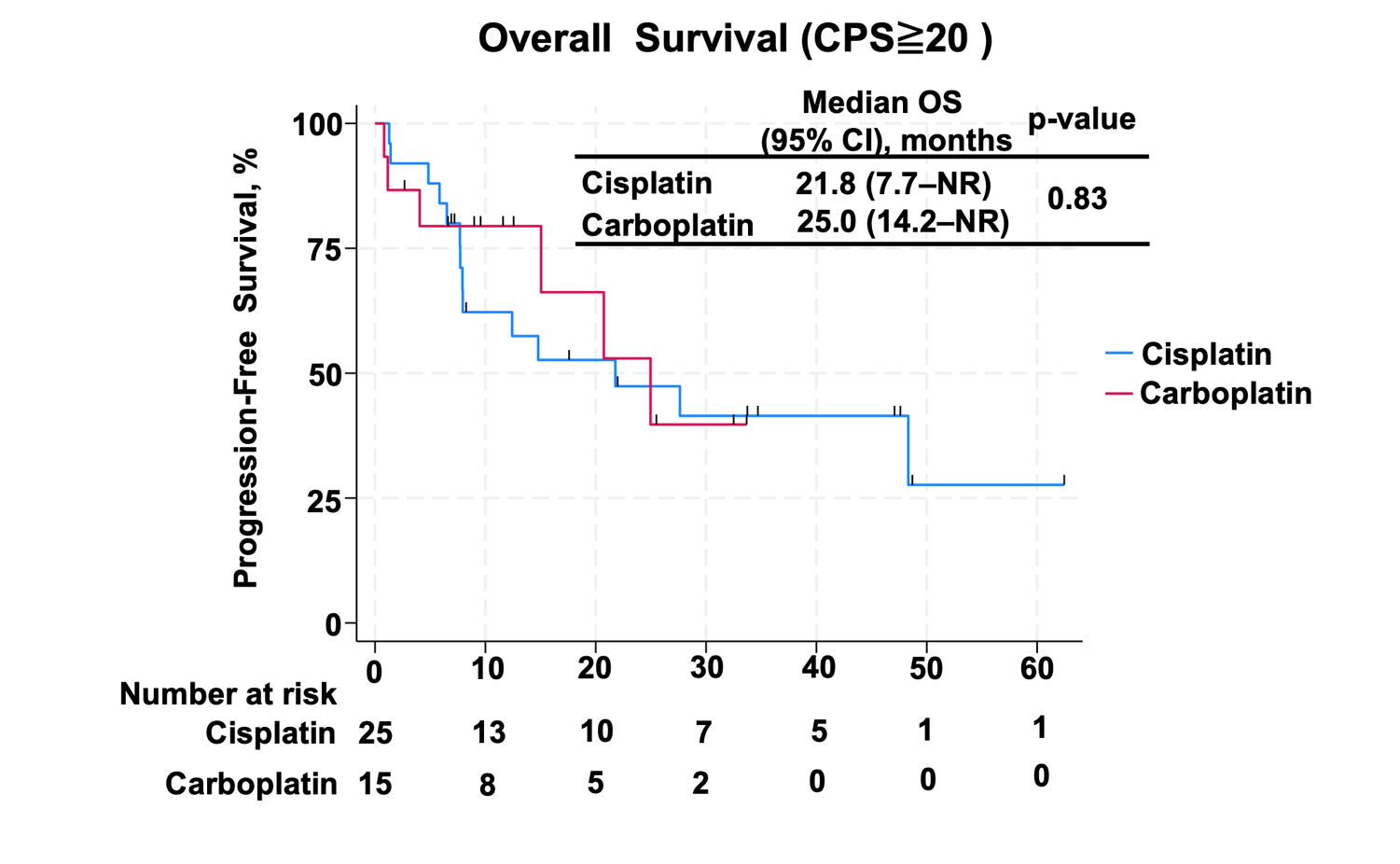
**

**Supplementary Figure 2.**

**Kaplan–Meier curves of PFS and OS by platinum regimen stratified by CPS.** (A) PFS (CPS 1–19); (B) PFS (CPS ≥20); (C) OS (CPS 1–19); (D) OS (CPS ≥20). Median survival (95% CI) and log-rank p-values are shown. Tick marks indicate censoring; numbers at risk are provided below.

**A.**

| **Multivariable sensitivity analysis for PFS** | | | | |
| --- | --- | --- | --- | --- |
| **Model** | **Variable** | **Hazard Ratio** | **95% CI** | **p-value** |
| **Main model** | Carboplatin vs Cisplatin | 0.70 | 0.40–1.23 | 0.22 |
| **With p16** | Carboplatin vs Cisplatin | 0.58 | 0.32–1.04 | 0.07 |
| **With primary tumor site** | Carboplatin vs Cisplatin | 0.63 | 0.34–1.17 | 0.14 |
| **With smoking history** | Carboplatin vs Cisplatin | 0.69 | 0.39–1.22 | 0.20 |
| **With age** | Carboplatin vs Cisplatin | 0.86 | 0.44–1.68 | 0.65 |
| **With ccr** | Carboplatin vs Cisplatin | 0.73 | 0.38–1.40 | 0.34 |
| **Truncated at 24 months** | Carboplatin vs Cisplatin | 0.69 | 0.39–1.22 | 0.20 |

**B.**

| **Multivariable sensitivity analysis for OS** | | | | |
| --- | --- | --- | --- | --- |
| **Model** | **Variable** | **Hazard Ratio** | **95% CI** | **p-value** |
| **Main model** | Carboplatin vs Cisplatin | 0.68 | 0.34–1.34 | 0.27 |
| **With p16** | Carboplatin vs Cisplatin | 0.63 | 0.30–1.34 | 0.23 |
| **With primary tumor site** | Carboplatin vs Cisplatin | 0.88 | 0.31–1.80 | 0.72 |
| **With smoking history** | Carboplatin vs Cisplatin | 0.64 | 0.32–1.25 | 0.19 |
| **With age** | Carboplatin vs Cisplatin | 0.67 | 0.30–1.49 | 0.32 |
| **With ccr** | Carboplatin vs Cisplatin | 0.45 | 0.20–1.02 | 0.06 |
| **Truncated at 24 months** | Carboplatin vs Cisplatin | 0.64 | 0.31–1.33 | 0.23 |

**Supplementary Table 1.**

Each model was adjusted for the PS and CPS classifications. Additional covariates were individually included to assess the robustness of the treatment effects of carboplatin versus cisplatin. The truncated models used the landmark time points of 24 months. Cisplatin was used as a reference for all comparisons. (A) Multivariable sensitivity analysis for PFS; (B) Multivariable sensitivity analysis for OS.

**A.**

|  | **All (N=76)** | **Cisplatin (N=41)** | **Carboplatin (N=35)** | **p-value** |
| --- | --- | --- | --- | --- |
| **G3≥Non-Hematological AEs, n (%)** | 28 (36.8) | 24 (58.5) | 4 (11.4) | **<0.01** |
| **Nausea, n (%)** | 9 (11.8) | 9 (22.0) | 0 (0.0) | **<0.01** |
| **Anorexia, n (%)** | 12 (15.8) | 12 (29.3) | 0 (0.0) | **<0.01** |
| **Malaise, n (%)** | 3 (3.9) | 3 (7.3) | 0 (0.0) | 0.25 |
| **Mucositis oral, n (%)** | 3 (3.9) | 3 (7.3) | 0 (0.0) | 0.25 |
| **Diarrhoea** **, n (%)** | 5 (6.6) | 5 (12.2) | 0 (0.0) | 0.06 |
| **Creatinine increased, n (%)** | 1 (1.3) | 1 (2.4) | 0 (0.0) | 1.00 |
| **ALT increased, n (%)** | 2 (2.6) | 2 (4.9) | 0 (0.0) | 0.50 |
| **AST increased, n (%)** | 1 (1.3) | 1 (2.4) | 0 (0.0) | 1.00 |
| **GGT increased, n (%)** | 3 (3.9) | 1 (2.4) | 2 (5.7) | 0.59 |
| **ALP increased, n (%)** | 1 (1.3) | 0 (0.0) | 1 (2.9) | 0.46 |
| **Hyponatremia, n (%)** | 3 (3.9) | 2 (4.9) | 1 (2.9) | 1.00 |
| **Hypokalemia, n (%)** | 2 (2.6) | 2 (4.9) | 0 (0.0) | 0.50 |
| **Hyperkalemia, n (%)** | 1 (1.3) | 0 (0.0) | 1 (2.9) | 0.46 |
| **Peripheral sensory neuropathy, n (%)** | 1 (1.3) | 1 (2.4) | 0 (0.0) | 1.00 |
| **Thromboembolic event, n (%)** | 1 (1.3) | 1 (2.4) | 0 (0.0) | 1.00 |
| **Pneumonitis, n (%)** | 1 (1.3) | 1 (2.4) | 0 (0.0) | 1.00 |
| **Adrenal insufficiency, n (%)** | 2 (2.6) | 2 (4.9) | 0 (0.0) | 0.50 |

**B.**

|  | **All (N=76)** | **Cisplatin (N=41)** | **Carboplatin (N=35)** | **p-value** |
| --- | --- | --- | --- | --- |
| **G2≥irAEs, n (%)** | 11 (13.2) | 8 (19.5) | 2 (5.7) | 0.10 |
| **Eczema, n (%)** | 5 (6.6) | 3 (7.3) | 2 (5.7) | 1.00 |
| **Adrenal insufficiency, n (%)** | 4 (5.3) | 3 (7.3) | 1 (2.9) | 0.62 |
| **Peripheral sensory neuropathy, n (%)** | 1 (1.3) | 1 (2.4) | 0 (0.0) | 1.00 |
| **Pneumonitis, n (%)** | 1 (1.3) | 1 (2.4) | 0 (0.0) | 1.00 |

**Supplementary Table 2.**

(A) Grade ≥3 non-haematological adverse events and (B) Grade ≥2 immune-related adverse events (irAEs) were assessed according to CTCAE v5.0. Comparisons between the cisplatin and carboplatin groups were performed using the chi-squared test or Fisher’s exact test, as appropriate.
